# Trend and forecast analysis of the changing disease burden of tuberculosis in China, 1990–2021

**DOI:** 10.1017/S0950268825100095

**Published:** 2025-07-15

**Authors:** Shun-Xian Zhang, Jin-Xin Zheng, Yu Wang, Wen-Wen Lv, Jian Yang, Ji-Chun Wang, Zhen-Hui Lu

**Affiliations:** 1 Longhua Hospital, Shanghai University of Traditional Chinese Medicine, Shanghai, 200032, China; 2 National Institute of Parasitic Diseases at Chinese Center for Disease Control and Prevention (Chinese Center for Tropical Diseases Research); NHC Key Laboratory of Parasite and Vector Biology; WHO Collaborating Centre for Tropical Diseases; National Center for International Research on Tropical Diseases; National Key Laboratory of Intelligent Tracking and Forecasting for Infectious Diseases, Shanghai, 200025, China; 3 School of Global Health, Chinese Center for Tropical Diseases Research-Shanghai Jiao Tong University School of Medicine, Shanghai 200025, China; 4 Clinical Research Institute, Shanghai Jiao Tong University School of Medicine, Shanghai 200025, China; 5 Department of Science and Technology, Chinese Center for Disease Control and Prevention, Beijing, 102206, China

**Keywords:** Bayesian age-period-cohort analysis, China, disease burden, epidemiology, tuberculosis

## Abstract

Tuberculosis (TB) remains a significant public health concern in China. Using data from the Global Burden of Disease (GBD) study 2021, we analyzed trends in age-standardized incidence rate (ASIR), prevalence rate (ASPR), mortality rate (ASMR), and disability-adjusted life years (DALYs) for TB from 1990 to 2021. Over this period, HIV-negative TB showed a marked decline in ASIR (AAPC = −2.34%, 95% CI: −2.39, −2.28) and ASMR (AAPC = −0.56%, 95% CI: −0.62, −0.59). Specifically, drug-susceptible TB (DS-TB) showed reductions in both ASIR and ASMR, while multidrug-resistant TB (MDR-TB) showed slight decreases. Conversely, extensively drug-resistant TB (XDR-TB) exhibited upward trends in both ASIR and ASMR. TB co-infected with HIV (HIV-DS-TB, HIV-MDR-TB, HIV-XDR-TB) showed increasing trends in recent years. The analysis also found an inverse correlation between ASIRs and ASMRs for HIV-negative TB and the Socio-Demographic Index (SDI). Projections from 2022 to 2035 suggest continued increases in ASIR and ASMR for XDR-TB, HIV-DS-TB, HIV-MDR-TB, and HIV-XDR-TB. The rising burden of XDR-TB and HIV-TB co-infections presents ongoing challenges for TB control in China. Targeted prevention and control strategies are urgently needed to mitigate this burden and further reduce TB-related morbidity and mortality.

## Introduction

Tuberculosis (TB), caused by *Mycobacterium tuberculosis* (Mtb), is an airborne infection primarily affecting the lungs and characterized by chronic systemic wasting [1–3]. Prolonged TB infection can cause extensive pulmonary and extrapulmonary damage, leading to various sequelae, complications, and comorbidities [4, 5]. Even in the absence of overt symptoms, individuals may experience impaired lung function, which increases all-cause mortality and reduces life expectancy. Moreover, TB imposes substantial physical and psychological burdens, diminishing patients’ quality of life [5].

Despite significant global efforts, TB remains a leading cause of morbidity and mortality worldwide [6, 7]. According to the 2024 World Health Organization (WHO) Global Tuberculosis Report, 10.8 million people developed TB in 2023 – an incidence of 134 per 100,000 population – of whom 55% were men, 33% were women, and 12% were children or adolescents. Among incident cases, 0.66 million (6.1%) involved co-infection with human immunodeficiency virus (HIV), and 0.40 million (3.7%) were classified as multidrug-resistant or rifampicin-resistant TB (MDR/RR-TB). In the same year, TB led to 1.25 million deaths, ranking it as the foremost cause of mortality from a single infectious agent – nearly double the deaths caused by acquired immunodeficiency syndrome (AIDS) [2].

Although TB is preventable and treatable with effective interventions, global progress in reducing its burden remains slow, with an annual decline in incidence of only 1.9% [2, 8]. Ongoing control efforts are hindered by the emergence of drug-resistant strains and enduring socio-economic barriers, including poverty, conflict, natural disasters, and the HIV epidemic [9]. In individuals living with HIV, the risk of active TB is approximately 15% within the first year of infection, and TB incidence increases twofold to threefold during this period [10].

China continues to face a high TB burden despite extensive research, substantial investment, and concerted government-led interventions that have contributed to a gradual decline in TB incidence and mortality [11]. However, this decline has been slower than anticipated, failing to meet established targets. Drawing on data from the Global Burden of Disease (GBD) Study 2021, the present study systematically examines trends in TB incidence, prevalence, mortality, and disability-adjusted life years (DALYs) in China from 1990 to 2021. The findings offer a robust evidence base for refining TB prevention and control strategies in China.

## Methods

### Definitions

Drug-susceptible tuberculosis (DS-TB) is defined as TB caused by *Mtb* strains that are sensitive to the standard first-line anti-TB drugs (isoniazid and rifampicin). MDR-TB without extensive drug resistance refers to strains resistant to at least isoniazid and rifampicin, but not to any fluoroquinolones (moxifloxacin, levofloxacin, or ofloxacin) or second-line injectable drugs (capreomycin, kanamycin, or amikacin). Extensively drug-resistant TB (XDR-TB) is characterized by strains resistant to isoniazid and rifampicin, in addition to at least one fluoroquinolone (moxifloxacin, levofloxacin, or ofloxacin) and at least one second-line injectable drug (capreomycin, kanamycin, or amikacin). HIV-DS-TB refers to TB caused by *Mtb* that is DS-TB in individuals co-infected with HIV. HIV-MDR-TB refers to MDR-TB in individuals co-infected with HIV, and HIV-XDR-TB denotes XDR-TB in individuals co-infected with HIV [6, 7, 12].

TB and its subtypes were defined using codes from the International Classification of Diseases, 10^th^ Revision (ICD-10: A10–A14, A15–A19.9, B90–B90.9, K67.3, K93.0, M49.0, N74.1, P37.0, U84.3) and ICD-9 (010–019.9, 137–137.9, 138.0, 138.9, 320.4, 730.4–730.6) in the GBD study 2021. HIV-TB co-infection was identified using the ICD-10 code B20.0 [13].

SDI, a composite metric reflecting socio-economic development, integrates three components: educational attainment among individuals aged ≥15 years, lag-distributed income per capita, and total fertility rate among women aged <25 years. SDI values (range: 0–1) categorize regions into five tiers: low (<0.46), low-middle (0.46–0.60), middle (0.61–0.69), high-middle (0.70–0.81), and high (>0.81) [13, 14].

### Data sources

Data were extracted from the GBD 2021 repository (https://ghdx.healthdata.org/), curated by the Institute for Health Metrics and Evaluation (IHME) at the University of Washington. This comprehensive database synthesizes 100,983 global disease-related data sources to estimate health burdens for 371 diseases and injuries across 204 countries and territories, alongside 88 risk factors [13].

For China, TB-specific metrics (1990–2021) included incidence, prevalence, mortality, and DALYs, reported as age-standardized rates (ASR) with 95% uncertainty intervals (UI), including age-standardized incidence rate (ASIR), prevalence rate (ASPR), mortality rate (ASMR), DALY rate, alongside absolute counts (cases, prevalence, deaths, DALYs). Estimates were generated using DisMod-MR 2.1 and meta-regression with Bayesian priors, regularization, and trimming (MR-BRT) models. Incidence and prevalence data were derived from China CDC surveillance systems, national health surveys, and peer-reviewed literature. Mortality estimates incorporated vital registration systems and census data. DALYs calculations integrated case reports, hospital discharge records, household surveys, and cohort studies [6, 7, 13].

### Statistical analysis

The disease burden of TB and its subtypes was evaluated using both rate-based metrics (incidence, prevalence, mortality, and DALYs, per 100,000 population) and absolute case counts. Rates quantify relative burden, while case counts reflect absolute burden. All estimates are reported with 95% *UI.* Detailed methodologies for percentage change (PC), estimated annual percentage change (EAPC), Joinpoint regression analysis, and Bayesian age-period-cohort (BAPC) modelling are described in subsequent sections [11, 15–17].

PC was calculated to quantify the proportional change in TB burden metrics (case numbers and ASR) between 1990 and 2021 using the formula [16–18]:

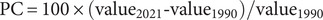



A positive trend was defined when the lower 95% UI bound of PC exceeded zero; a negative trend was inferred if the upper 95% UI bound fell below zero [15].

EAPC was computed using regression models to estimate the annual rate of change in ASR (ASIR, ASPR, ASMR, DALY rate) from 1990 to 2021. Analyses were performed in R software (version 4.3.1) with α = 0.05 significance thresholds. The model structure follows [6, 7]:

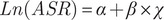



In the equation, *α* is the intercept, *β* is the slope, *χ* is year. An EAPC with 95% confidence interval (*CI*) entirely above zero indicates an upward trend; entirely below zero signifies a decline [16, 17].

### Joinpoint regression analysis

Average annual percentage change (AAPC) and annual percentage change (APC) were derived using Joinpoint Regression Software (v5.1.0) to quantify temporal trends in TB burden metrics (ASIR, ASPR, ASMR, Age-standardized DALY rate) from 1990 to 2021 and from 2022 to 2035. The segmented linear regression model is defined as [11, 19, 20]:

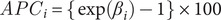




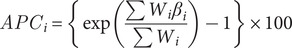



In this model, the parameter *i* represents the number of segments, while *β_i_* corresponds to the regression coefficients for each linear segment of the data. *W_i_* is the regression coefficient weights determined by the length of each corresponding segment. Trends were classified as increasing (AAPC/APC 95% *CI* > 0) or decreasing (AAPC/APC 95% *CI* < 0) [11, 19, 20].

### BAPC models

BAPC models projected age-standardized incidence and mortality rates for TB subtypes in China (2022–2035) using the framework [19, 21]:

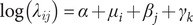



In the model, *i* (1≤*i*≤*I*) represents the time points, *j* (1≤*i*≤*J*) represents the age groups, *α* denotes the intercept, *μ_i_* signifies the age effect, *β_j_* represents the period effect, *γ_k_* indicates the cohort effect [19]. Model selection employed Monte Carlo permutation tests and Bayesian information criteria [22].

### Correlation analysis

Spline smoothing models assessed associations between TB subtype age-standardized rates (ASIR, ASPR, ASMR, Age-standardized DALY rate) and the SDI. Locally weighted scatterplot smoothing determined knot placement and polynomial degrees. Statistical significance was defined as p < 0.05 [14, 16].

## Results

### ASIR

In 2021, HIV-negative individuals in China exhibited an ASIR of 36.28 per 100,000 population (95% *UI*: 32.6, 40.40) for TB, comprising DS-TB (34.65 per 100,000 population, 95%*UI*: 29.43, 39.39), MDR-TB (1.49 per 100,000 population, 95%*UI*: 0.25, 4.66), and XDR-TB (0.13 per 100,000 population, 95%*UI*: 0.02, 0.41). Among HIV-TB co-infection, ASIRs for HIV-DS-TB (1.17 per 100,000 population, 95%*UI*: 0.98, 1.33), HIV-MDR-TB (0.06 per 100,000 population, 95%*UI*: 0.01, 0.19), and HIV-XDR-TB (0.01 per 100,000 population, 95%*UI*: 0.01, 0.02) were substantially low (Table 1). Temporal trends from 1990 to 2021 revealed notable declines in ASIR for HIV-negative TB (AAPC = −2.34%, 95%*CI*: −2.39, −2.28), DS-TB (AAPC = −2.26%, 95%*CI*: −2.32, −2.20), and MDR-TB (AAPC = −0.08%, 95%*CI*: −0.09, −0.07). Conversely, emerging upward trajectories were observed for XDR-TB (AAPC = 0.01%, 95%*CI*: 0.00, 0.01), HIV-DS-TB (AAPC = 0.02%, 95%*CI*: 0.01, 0.02), HIV-MDR-TB (AAPC = 0.01%, 95%*CI*: 0.00, 0.01), and HIV-XDR-TB (AAPC = 0.01%, 95%*CI*: 0.00, 0.01) (Table 2).

**Table 1.** ASR of tuberculosis and its subtypes in 2021, and the EAPC of ASR were analysed in China, 1990–2021

Disease	Incidence		Prevalence		Death		DALYs	
	ASIR (per 100,000 population, 95%*UI*). 2021.	EAPC (95% *CI*). 1990–2019.	ASIR (per 100,000 population, 95%*UI*). 2021.	EAPC (95% *CI*). 1990–2019.	ASMR (per 100,000 population, 95%*UI*). 2021.	EAPC (95% *CI*). 1990–2019.	ASIR (per 100,000 population, 95%*UI*). 2021.	EAPC (95% *CI*). 1990–2019.
TB	36.28(32.63, 40.47)	−3.78(−3.90, −3.66)	30,557.45(27,692.69, 33,531.31)	−0.40(−0.53, −0.27)	1.91(1.51, 2.51)	−7.79(−8.07, −7.30)	76.22(62.59, 94.45)	−7.49(−7.71, −7.26)
Male	46.75(41.84, 51.91)	−3.49(−3.60, −3.39)	31,679.16(28,788.98, 34,672.50)	−0.38(−0.51, −0.26)	2.90(2.13, 4.12)	−7.19(−7.47, −6.90)	108.66(85.47, 145.29)	−6.84(−7.04, −6.64)
Female	26.27(23.19, 29.49)	−4.35(−4.40, −4.11)	29,399.21(26,567.98, 32,460.33)	−0.42(−0.56, −0.28)	1.03(0.79, 1.37)	−8.99(−9.32, −8.66)	45.02(36.46, 55.79)	−8.66(−8.96, −8.37)
DS-TB	34.65(29.43, 39.39)	−3.58(−3.70, −3.47)	69.86(57.49, 79.25)	−3.12(−3.22, −3.02)	1.73(1.23, 2.29)	−7.51(−7.75, −7.28)	70.21(53.51, 86.29)	−7.19(−7.38, −7.00)
Male	44.65(38.17, 50.51)	−3.30(−3.40, −3.19)	93.9(77.52, 106.96)	−2.97(−3.06, −2.88)	2.63(1.80, 3.86)	−6.91(−7.14, −6.68)	99.99(73.47, 131.5)	−6.55(−6.72, −6.39)
Female	25.1(21.04, 28.68)	−4.05(−4.18, −3.92)	47.51(39.19, 54.6)	−3.42(−3.56, −3.27)	0.93(0.66, 1.27)	−8.72(−9.01, −8.44)	41.58(31.01, 53.02)	−8.36(−8.61, −8.11)
MDR-TB	1.49(0.25, 4.66)	−6.60(−7.85, −5.33)	2.98(0.5, 9.38)	−5.52(−6.81, −4.21)	0.15(0.02, 0.43)	−8.06(−9.26, −6.84)	5.11(0.90, 15.03)	−10.07(−11.29, −8.84)
Male	1.93(0.33, 6.02)	−6.30(−7.55, −5.03)	4.01(0.68, 12.64)	5.37(−6.68, −4.04)	0.22(0.04, 0.67)	−7.42(−8.63, −6.18)	7.36(1.28, 22.04)	−9.39(−10.61, −8.16)
Female	1.08(0.18, 3.45)	−7.09(−8.35, −5.81)	2.02(0.33, 6.41)	−5.82(−7.07, −4.55)	0.08(0.01, 0.24)	−9.33(−10.53, −8.11)	2.93(0.48, 9.11)	−11.32(−12.56, −10.07)
XDR-TB	0.13(0.02, 0.41)	2.04(−0.31, 4.44)	0.26(0.04, 0.82)	3.50(1.26, 5.79)	0.03(0.01, 0.09)	−2.89(−5.04, −0.69)	0.9(0.14, 2.75)	3.19(−2.35, 9.05)
Male	0.17(0.03, 0.53)	2.37(−0.02, 4.81)	0.35(0.06, 1.11)	3.67(1.39, 6.00)	0.04(0.01, 0.14)	−2.25(−4.45, 0.01)	1.31(0.20, 4.23)	3.93(−1.59, 9.75)
Female	0.09(0.02, 0.3)	1.49(−0.82, 3.84)	0.18(0.03, 0.56)	3.18(1.02, 5.38)	0.02(0.01, 0.05)	−4.20(−6.32, −2.03)	0.50(0.07, 1.56)	1.76(−3.84, 7.68)
HIV-DS-TB	1.17(0.98, 1.33)	1.35(0.88, 1.83)	2.43(1.99, 2.82)	2.44(1.81, 3.08)	0.16(0.10, 0.25)	2.09(0.91, 3.28)	8.48(5.36, 12.26)	2.47(1.32, 3.63)
Male	1.72(1.45, 1.96)	1.64(1.20, 2.08)	3.57(2.94, 4.14)	2.74(2.12, 3.35)	0.24(0.14, 0.37)	2.30(1.18, 3.44)	12.27(7.73, 17.77)	2.77(1.65, 3.90)
Female	0.6(0.51, 0.69)	0.58(0.06, 1.10)	1.26(1.03, 1.47)	1.68(1.01, 2.36)	0.08(0.05, 0.12)	1.43(0.14, 2.74)	4.56(2.86, 6.57)	1.77(0.55, 3.01)
HIV-MDR-TB	0.06(0.01, 0.19)	−2.16(−3.81, −0.48)	0.13(0.02, 0.39)	−0.32(−2.17, 1.55)	0.02(0.01, 0.05)	−0.75(−3.17, 1.73)	0.8(0.12, 2.47)	−0.37(−2.78, 2.10)
Male	0.09(0.01, 0.28)	−1.91(−3.52, −0.27)	0.18(0.03, 0.58)	−0.03(−1.85, 1.82)	0.02(0.01, 0.08)	−0.54(−2.92, 1.89)	1.15(0.17, 3.55)	−0.07(−2.46, 2.37)
Female	0.03(0.01, 0.1)	−2.88(−4.58, −1.14)	0.07(0.01, 0.2)	−1.07(−2.95, 0.84)	0.01(0.00, 0.03)	−1.39(−3.91, 1.19)	0.43(0.07, 1.34)	−1.04(−3.51, 1.48)
HIV-XDR-TB	0.01(0.00, 0.02)	7.20(4.16, 10.34)	0.01(0.00, 0.03)	9.25(6.17, 12.42)	0.01(0.00, 0.02)	5.65(2.44, 8.95)	0.15(0.02, 0.52)	13.92(6.87, 21.43)
Male	0.01(0.00, 0.03)	7.48(4.48, 10.56)	0.02(0.01, 0.05)	9.58(6.52, 12.74)	0.01(0.00, 0.02)	5.92(2.77, 9.17)	0.22(0.03, 0.74)	14.23(7.22, 21.69)
Female	0.01(0.00, 0.01)	6.34(3.27, 9.51)	0.01(0.01, 0.05)	8.36(5.30, 11.52)	0.01(0.00, 0.02)	4.83(1.55, 8.22)	0.08(0.01, 0.29)	13.13(6.05, 20.67)

Abbreviations: ASIR: age-standardized incidence rate. ASMR: age-standardized mortality rate. ASPR: age-standardized prevalence rate. ASR: age-standardized rate. *CI*: confidence interval. DALYs: Disability-adjusted life years. DS-TB: drug-susceptible tuberculosis. EAPC: estimated annual percentage change. HIV: human immunodeficiency virus. HIV-DS-TB: HIV-infected drug-susceptible tuberculosis. HIV-MDR-TB: HIV-infected multidrug-resistant tuberculosis without extensive drug resistance. HIV-XDR-TB: HIV-infected extensively drug-resistant tuberculosis. MDR-TB: multidrug-resistant tuberculosis without extensive drug resistance. TB: Tuberculosis. UI: uncertainty interval. XDR-TB: extensively drug-resistant tuberculosis.

**Table 2.** Analysis of trends in the burden of TB and its subtypes in China, 1990–2021

Disease	Index	ASIR		ASPR		ASMR		Age-standardized DALY rate	
		Years	Change(%, 95% *CI*)	Years	Change(%, 95% *CI*)	Years	Change(%, 95% *CI*)	Years	Change(%, 95% *CI*)
TB	AAPC	1990–2021	−2.34(−2.39, −2.28)	1990–2021	−27.18(−53.17, −1.18)	1990–2021	−0.56(−0.62, −0.59)	1990–2021	−20.95(−21.22, −20.68)
	APC	1990–1995	−2.00(−2.97, −1.03)	1990–1999	0.44(0.07, 0.82)	1990–1998	−7.30(−7.69, −6.91)	1990–1998	−6.40(−6.54, −6.26)
	APC	1995–2002	−4.58(−5.52, −3.63)	1999–2004	−1.71(−2.38, −1.03)	1998–2004	−4.40(−5.38, −3.40)	1998–2004	−11.31(−12.28, −10.32)
	APC	2002–2016	−3.91(−4.17, −3.64)	2004–2016	−0.48(−0.75, −0.20)	2004–2010	−12.04(−12.59, −11.50)	2004–2010	−7.89(−8.61, −7.17)
	APC	2016–2021	−2.26(−3.22, −1.29)	2016–2021	1.56(0.86, 2.26)	2010–2021	−6.14(−6.43,–5.85)	2010–2021	−5.12(−5.48, −4.76)
HIV	AAPC	1990–2021	0.05(0.047,0.055)	1990–2021	0.90(0.88, 0.91)	1990–2021	0.06(0.05, 0.06)	1990–2021	2.63(2.53, 2.73)
	APC	1990–1992	23.60(9.99, 38.89)	1990–2005	13.23(13.08, 13.38)	1990–1994	13.23(13.08, 13.38)	1990–1994	21.23(16.53, 26.12)
	APC	1992–2003	14.53(13.75, 15.33)	2005–2008	6.44(5.39, 7.51)	1994–2003	6.44(5.39, 7.51)	1994–2004	13.05(11.24, 14.89)
	APC	2003–2014	−7.01(−7.85, −6.16)	2008–2013	1.62(0.91, 2.33)	2003–2011	1.62(0.91, 2.33)	2004–2011	0.51(−1.42, 2.45)
	APC	2014–2021	0.24(−1.03, 1.52)	2013–2021	−1.46(−1.79, −1.12)	2011–2021	−1.46(−1.79, −1.12)	2011–2021	6.54(5.08, 8.01)
DS-TB	AAPC	1990–2021	−2.26(−2.32, −2.20)	1990–2021	−3.70(−3.78, −3.62)	1990–2021	−0.55(−0.57, −0.54)	1990–2021	−19.83(−20.08, −19.58)
	APC	1990–2005	−4.14(−4.23, −4.04)	1990–1997	−2.40(−2.92,–1.88)	1990–1997	−9.40(−9.78, −9.01)	1990–1997	−8.87(−9.31, −8.43)
	APC	2005–2011	−1.79(−2.35, −1.22)	1997–2005	−4.21(−4.83, −3.58)	1997–2004	−4.18(−4.81, −3.55)	1997–2004	−5.44(−5.91, −4.98)
	APC	2011–2014	−5.95(−6.72, −5.18)	2005–2009	−1.20(−2.71, 0.34)	2004–2009	−11.61(−12.19, −11.02)	2004–2010	−9.93(−10.48, −9.37)
	APC	2014–2021	−2.07(−2.40, −1.73)	2009–2021	−3.44(−3.69, −3.19)	2009–2021	−6.10(−6.32, −5.88)	2010–2021	−5.63(−5.84, −5.43)
MDR-TB	AAPC	1990–2021	−0.08(−0.09, −0.07)	1990–2021	−0.06(−0.08, −0.05)	1990–2021	−0.04(−0.04, −0.03)	1990–2021	−1.23(−1.35, −1.11)
	APC	1990–1993	38.52(32.75, 44.54)	1990–1993	42.32(35.99, 48.93)	1990–1993	27.83(23.94, 31.84)	1990–1993	28.30(24.24, 32.49)
	APC	1993–2003	−4.62(−5.79, −3.44)	1993–2002	−3.18(−4.44, −1.90)	1993–2004	−5.95(−6.66, −5.23)	1993–2003	−6.68(−7.42, −5.94)
	APC	2003–2015	−11.76(−12.46, −11.06)	2002–2015	−10.36(−11.03, −9.68)	2004–2014	−17.42(−18.05, −16.79)	2003–2014	−16.80(−17.46, −16.14)
	APC	2015–2021	−0.62(−2.39,1.18)	2015–2021	−1.03(−3.41, 1.40)	2014–2021	−5.42(−6.43, −4.41)	2014–2021	−5.00(−6.04, −3.94)
XDR-TB	AAPC	1990–2021	0.01(0.00,0.01)	1990–2021	0.01(0.01, 0.02)	1990–2021	0.01(0.00, 0.01)	1990–2021	0.04(0.03, 0.04)
	APC	1990–1996	49.34(43.79, 55.09)	1990–1993	79.64(59.49, 102.33)	1990–1995	76.44(66.74, 86.69)	1990–1993	723.92(472.02, 1,086.63)
	APC	1996–2004	5.96(3.41, 8.58)	1993–2000	22.90(18.06, 27.95)	1995–2003	8.12(6.79, 9.46)	1993–1996	39.33(−3.26, 100.66)
	APC	2004–2015	−7.65(−9.24, −6.02)	2000–2016	−3.01(−3.81, −2.20)	2003–2014	−11.95(−12.72, −11.17)	1996–2002	8.00(−8.27, 27.13)
	APC	2015–2021	3.20(0.16, 6.34)	2016–2021	2.58(−2.74, 8.18)	2014–2021	−2.17(−3.37, −0.95)	2092–2021	−8.12(−9.94, −6.26)
HIV-DS-TB	AAPC	1990–2021	0.02(0.01, 0.02)	1990–2021	0.05(0.04, 0.05)	1990–2021	0.01(0.00, 0.01)	1990–2021	0.20(0.18, 0.22)
	APC	1990–1996	7.51(6.58, 8.44)	1990–1998	9.42(8.78, 10.06)	1990–2002	11.81(11.01, 12.61)	1990–2003	11.72(11.08, 12.37)
	APC	1996–2004	3.19(2.61, 3.76)	1998–2004	3.71(2.97, 4.46)	2002–2013	−5.372(−6.37, −4.36)	2003–2013	−4.19(−5.00, −3.37)
	APC	2004–2013	−1.43(−1.89, −0.96)	2004–2012	−0.84(−1.55, −0.13)	2013–2016	9.27(2.06, 16.70)	2013–2016	6.25(0.59, 12.23)
	APC	2013–2021	1.22(0.75, 1.69)	2012–2021	1.02(0.60,1.44)	2016–2021	−3.61(−5.81, −1.36)	2016–2021	−3.08(−4.85, −1.27)
HIV-MDR-TB	AAPC	1990–2021	0.01(0.00, 0.01)	1990–2021	0.01(0.00, 0.01)	1990–2021	0.01(0.00, 0.01)	1990–2021	0.02(0.02, 0.03)
	APC	1990–1993	57.63(48.79, 67.00)	1990–1992	87.84(70.53,106.91)	1990–1994	56.78(50.13, 63.73)	1990–1994	65.91(57.28, 75.01)
	APC	1993–2004	1.92(0.48, 3.38)	1992–2002	6.99(5.39, 8.61)	1994–2004	7.83(5.94, 9.76)	1994–2003	9.65(8.21,11.10)
	APC	2004–2014	−10.12(−11.22, −9.00)	2002–2015	−7.95(−8.99, −6.89)	2004–2012	−13.82(−15.33, −12.28)	2003–2013	−12.82(−14.15, −11.47)
	APC	2014–2021	2.38(−0.09, 4.91)	2015–2021	2.23(−0.38, 4.91)	2012–2021	−0.58(−2.33, 1.20)	2013–2021	−1.03(−2.54, 0.51)
HIV-XDR-TB	AAPC	1990–2021	0.01(0.00, 0.01)	1990–2021	0.01(0.00, 0.01)	1990–2021	0.01(0.00, 0.01)	1990–2021	0.01(0.01, 0.02)
	APC	1990–1995	70.78(61.84, 80.22)	1990–1994	99.43(83.29,116.99)	1990–1996	95.72(82.06, 110.40)	1990–1994	897.42(609.08, 1,302.86)
	APC	1995–2003	21.51(17.68, 25.48)	1994–2002	24.82(21.23, 28.50)	1996–2004	21.57(17.91, 25.35)	1994–2002	34.47(26.35, 43.12)
	APC	2003–2015	−5.14(−6.67, −3.59)	2002–2015	−1.73(−3.27, −0.16)	2004–2012	−7.94(−9.84, −5.99)	2002–2013	−5.93(−10.16,–1.50)
	AOC	2015–2021	4.86(2.15, 7.65)	2015–2021	6.66(2.93,10.54)	2012–2021	3.14(1.00, 5.31)	2013–2021	2.43(−4.92, 10.35)

Abbreviations: AAPC: Average Annual Percent Change. APC: Annual Percent Change. ASIR: age-standardized incidence rate. ASPR: age-standardized prevalence rate. ASMR: age-standardized mortality rate. CI: confidence interval. DALYs: Disability-adjusted life years. DS-TB: drug-susceptible tuberculosis. HIV: human immunodeficiency virus. HIV-DS-TB: HIV-infected drug-susceptible tuberculosis. HIV-MDR-TB: HIV-infected multidrug-resistant tuberculosis without extensive drug resistance. HIV-XDR-TB: HIV-infected extensively drug-resistant tuberculosis. MDR-TB: multidrug-resistant tuberculosis without extensive drug resistance. TB: Tuberculosis. XDR-TB: extensively drug-resistant tuberculosis.

### ASPR

For HIV-negative individuals in 2021, ASPRs were 68.86 per 100,000 population (95%*UI*: 57.49, 79.25) for DS-TB, 2.98 per 100,000 population (95%*UI*: 0.50, 9.38) for MDR-TB, and 0.26 per 100,000 population (95%*UI*: 0.04, 0.82) for XDR-TB. HIV-TB co-infection subtypes showed low disease burdens: HIV-DS-TB (2.43 per 100,000 population, 95%UI: 1.99, 2.82), HIV-MDR-TB (0.13 per 100,000 population, 95%*UI*: 0.02, 0.39), and HIV-XDR-TB (0.01 per 100,000 population, 95%*UI*: 0.00, 0.03) (Table 1). Longitudinal analysis identified declining ASPRs for HIV-negative TB (AAPC = −27.18%, 95%*CI*: −53.17, −1.18), DS-TB (AAPC = −3.70%, 95%*CI*: −3.78, −3.62), and MDR-TB (AAPC = −0.06%, 95%*CI*: −0.08, −0.05). In contrast, rising trends were documented for XDR-TB (AAPC = 0.01%, 95%*CI*: 0.01, 0.02), HIV-DS-TB (AAPC = 0.05%, 95%*CI*: 0.04, 0.05), HIV-MDR-TB (AAPC = 0.01%, 95%CI: 0.00, 0.01), and HIV-XDR-TB (AAPC = 0.01%, 95%*CI*: 0.00, 0.01) (Table 2).

### ASMR

HIV-negative individuals in 2021 demonstrated an ASMR of 1.19 per 100,000 population (95%*UI*: 1.51, 2.51) for TB, with subtype-specific rates of 1.73 per 100,000 population (95%UI: 1.23, 2.29) for DS-TB, 0.15 per 100,000 population (95%*UI*: 0.02, 0.43) for MDR-TB, and 0.03 per 100,000 population (95%*UI*: 0.01, 0.09) for XDR-TB. HIV-associated subtypes showed low mortality burdens: HIV-DS-TB (0.16 per 100,000 population, 95%*UI*: 0.10, 0.25), HIV-MDR-TB (0.02 per 100,000 population, 95%*UI*: 0.01, 0.05), and HIV-XDR-TB (0.01 per 100,000 population, 95%*UI*: 0.00, 0.02) (Table 1). Mortality trends from 1990 to 2021 showed declines for HIV-negative TB (AAPC = −0.56%, 95%*CI*: −0.62, −0.59), DS-TB (AAPC = −0.55%, 95%*CI*: −0.57, −0.54]), and MDR-TB (AAPC = −0.04%, 95%*CI:* −0.04, −0.03). Conversely, rising mortality trajectories were observed for XDR-TB (AAPC = 0.01%, 95%*CI*: 0.00, 0.01), HIV-DS-TB (AAPC = 0.01% 95%*CI*: 0.00, 0.01), HIV-MDR-TB (AAPC = 0.01%, 95%*CI*: 0.00, 0.01), and HIV-XDR-TB (AAPC = 0.01%, 95%*CI*: 0.00, 0.01) (Table 2).

### Age-standardized DALY rate

In 2021, HIV-negative individuals experienced a TB-associated DALY rate of 76.22 per 100,000 population (95%*UI*: 62.59, 94.45), driven by DS-TB (70.21 per 100,000 population, 95%*UI*: 53.51, 86.29), MDR-TB (5.11 per 100,000 population, 95%*UI*: 0.90, 15.03), and XDR-TB (0.90 per 100, 000 population, 95%*UI*: 0.14, 2.75). HIV-associated subtypes contributed additional burdens, HIV-DS-TB (8.48 per 100,000 population, 95%*UI*: 5.36, 12.26), HIV-MDR-TB (0.80 per 100,000 population, 95%*UI*: 0.12, 2.47), and HIV-XDR-TB (0.15 per 100,000 population, 95%*UI*: 0.02, 0.52) (Table 1). Temporal analysis revealed declining DALY rates for HIV-negative TB (AAPC = −20.95%, 95%*CI*: −21.22, −20.68), DS-TB (AAPC = −19.83%, 95%*CI*: −20.08, −19.58), and MDR-TB (AAPC = −1.23%, 95%*CI*: −1.35, −1.11). However, upward trends were observed for XDR-TB (AAPC = 0.04%, 95%*CI*: 0.03, 0.04), HIV-DS-TB (AAPC = 0.20%, 95%*CI*: 0.18. 0.22), HIV-MDR-TB (AAPC = 0.02%, 95%*CI*: 0.02, 0.03), and HIV-XDR-TB (AAPC = 0.01%, 95%*CI*: 0.01, 0.02) (Table 2).

### Age-group distribution

In 2021, HIV-negative males exhibited higher incidence rates of total TB and DS-TB compared to females, with pronounced disparities emerging in males aged ≥40–44 years. Similarly, HIV-DS-TB incidence rate showed male predominance, particularly among those aged ≥20–24 years (Supplementary Figure S1 A–G).

The analyses revealed elevated TB rates among HIV-negative males, with DS-TB prevalence disproportionately affecting males aged ≥50–54 years. Among HIV-positive individuals, HIV-DS-TB prevalence remained higher in males aged ≥45–49 years relative to females (Supplementary Figure S2 A–G).

Mortality patterns mirrored these trends: HIV-negative males demonstrated higher TB and DS-TB mortality rates, with excess TB deaths observed from age ≥ 20–24 years and DS-TB mortality differentials emerging from age ≥ 25–29 years. HIV-DS-TB mortality showed similar male predominance, with significant disparities persisting in males aged ≥30–34 years (Supplementary Figure S3 A–G).

DALY rates further underscored this gender imbalance. HIV-negative males experienced higher TB- and DS-TB-associated DALYs, with disparities widening from age ≥ 30–34 years. HIV-DS-TB DALY rates also disproportionately burdened males aged ≥20–24 years (Supplementary Figure S4 A–G).

### The correlation between ASR and SDI

The analysis revealed robust inverse associations between SDI and ASIR of HIV-negative TB (*r* = −0.999, *p* < 0.001), DS-TB (*r* = −0.998, *p* < 0.001), and MDR-TB (*r* = −0.868, *p* < 0.001). Conversely, ASIRs of HIV-DS-TB (*r* = 0.642, *p* < 0.001) and HIV-XDR-TB (*r* = 0.508, *p* = 0.001) demonstrated significant positive correlations with SDI (Supplementary Table S1).

The analysis revealed robust inverse associations between SDI and ASPR of TB (*r* = −0.696, *p* < 0.001), DS-TB (*r* = −0.999, *p* < 0.001), and MDR-TB (*r* = −0.835, *p* < 0.001). No significant correlation was observed between SDI and ASPR of XDR-TB (*r* = 0.215, *p* = 0.244). In contrast, ASPRs of HIV-DS-TB (*r* = 0.848, *p* < 0.001) and HIV-XDR-TB (*r* = 0.793, *p* < 0.001) exhibited strong positive correlations with SDI (Supplementary Table S1).

The ASMR of TB (*r* = −0.999, *p* < 0.001), DS-TB (*r* = −0.998, *p* < 0.001), and MDR-TB (*r* = −0.937, *p* < 0.001) demonstrated pronounced negative correlations with SDI. Conversely, ASMRs of HIV-DS-TB (*r* = 0.374, *p* = 0.004) and HIV-XDR-TB (*r* = 0.521, *p* = 0.002) showed significant positive associations with SDI (Supplementary Table S1).

The age-standardized DALY rate of TB (*r* = −0.999, *p* < 0.001), DS-TB (*r* = −0.999, *p* < 0.001), and MDR-TB (*r* = −0.937, *p* < 0.001) showed a significant negative correlation with the SDI. In contrast, the age-standardized DALY rate for HIV-DS-TB (*r* = 0.424, *p* = 0.016) and HIV-XDR-TB (*r* = 0.464, *p* = 0.009) were positively correlated with the SDI (Supplementary Table S1).

### Projecting ASIR and ASMR

Projections from the BAPC model indicate a sustained decline in ASIR and ASMR for HIV-negative TB, DS-TB, and MDR-TB between 2022 and 2035. In contrast, ASIR and ASMR for XDR-TB, HIV-DS-TB, HIV-MDR-TB, and HIV-XDR-TB are projected to exhibit a steady upward trajectory (Table 3).

**Table 3.** Predicted ASIR and ASMR of TB and subtypes spanning 2022–2035 in China, based on the BAPC model

Disease	ASIR(per 100,000 population, 95%*CI*). 2035 year	AAPC of ASIR (%,95%*CI*) 2022–2035	ASMR(per 100,000 population, 95%*CI*). 2035 year	AAPC of ASMR (%,95% *CI*) 2022–2035. year
TB	20.29(17.17, 23.40)	−1.07(−1.07, −1.06)	0.63(0.46, 0.80)	−0.09(−0.09, −0.09)
DS-TB	19.34(16.52, 22.17)	−1.02(−1.03, −1.02)	0.57(0.42, 0.71)	−0.08(−0.08, −0.08)
MDR-TB	0.96(0.01, 2.11)	−0.04(−0.03, −0.05)	0.06(0.01, 0.12)	−0.01(−0.02, −0.01)
XDR-TB	0.78(0.01, 4.38)	0.05(0.04, 0.06)	0.19(0.01, 1.27)	0.01(0.01, 0.02)
HIV-DS-TB	1.78(1.15, 2.40)	0.04(0.04, 0.05)	0.31(0.06, 0.56)	0.01(0.01, 0.02)
HIV-MDR-TB	0.09(0.01, 0.23)	0.01(0.01, 0.02)	0.03(0.01, 0.09)	0.01(0.01, 0.02)
HIV-XDR-TB	0.02(0.01, 0.07)	0.01(0.01, 0.01)	0.02(0.01, 0.05)	0.01(0.01, 0.02)

*Notes*: When the ASIR and ASMR is predicted for a given year, if the lower limit of the 95% CIs is below 0, 0 is set. Abbreviation: ASIR: age-standardized incidence rate. ASMR, age-standardized mortality rate. BAPC: Bayesian age-period-cohort. CI: confidence interval. DS-TB: drug-susceptible tuberculosis. EAPC: estimated annual percentage change. HIV: human immunodeficiency virus. HIV-DS-TB: HIV-infected drug-susceptible tuberculosis. HIV-MDR-TB: HIV-infected multidrug-resistant tuberculosis without extensive drug resistance. HIV-XDR-TB: HIV-infected extensively drug-resistant tuberculosis. MDR-TB: multidrug-resistant tuberculosis without extensive drug resistance. TB: Tuberculosis. UI: uncertainty interval. XDR-TB: extensively drug-resistant tuberculosis.

## Discussion

This study reveals that while China has achieved a sustained decline in the ASIR and ASMR of TB over the past three decades, recent years have witnessed concerning increases in ASIR associated with XDR-TB and HIV-TB co-infection. These emerging epidemiological patterns pose substantial challenges to national TB containment efforts. The findings underscore the urgent need to refine existing TB control strategies through multisectoral innovations. Critical priorities include advancing next-generation diagnostic technologies, accelerating vaccine development, and optimizing therapeutic regimens through novel antimicrobial agents. Such targeted innovations are essential to effectively mitigate the persistent public health threat posed by TB and its drug-resistant variants, while addressing disparities in healthcare access and pathogen adaptation dynamics.

The findings demonstrate that while TB burden has declined in both sexes, males exhibit consistently higher disease metrics across all indicators – including incidence, mortality, prevalence, and DALYs – compared to females. Notably, HIV-negative males aged over 24 years maintained elevated TB mortality rates relative to their female counterparts across all SDI strata globally, a pattern corroborated by prior studies [6, 7]. Epidemiological data reveal that HIV-negative males in numerous countries experience approximately 50% higher TB incidence rates and nearly double the mortality rates observed in females [6, 7]. These findings collectively suggest entrenched biological and gender-related socio-economic determinants driving disproportionate TB outcomes in male populations. This disparity can be attributed to several contributing factors. Men are often subjected to greater societal pressures and are more likely to engage in health-compromising behaviours, such as smoking and excessive alcohol consumption, which are established risk factors for both the acquisition and exacerbation of TB [23]. This observed gender disparity underscores the necessity of incorporating gender-specific considerations into the design and implementation of public health interventions aimed at TB control [24]. For instance, promoting healthier lifestyle choices among men, including smoking cessation and moderation of alcohol intake, could enhance their resilience against TB and ultimately alleviate the overall disease burden [23].

The study demonstrates that TB incidence and mortality among individuals aged 65 years and older in China exhibit a progressive increase with advancing age. This phenomenon may be driven by a combination of physiological and sociostructural factors specific to ageing populations [1]. Age-related decline in immune function heightens susceptibility to *Mtb* infection, elevates the likelihood of rapid disease progression following infection, and amplifies risks of clinical deterioration [25]. Furthermore, comorbidities such as diabetes mellitus and chronic obstructive pulmonary disease, prevalent in older adults, compromise immune regulation, reduce host resilience, and exacerbate both the clinical trajectory of TB and therapeutic challenges [26]. Atypical presentations of TB in elderly patients frequently contribute to diagnostic delays, resulting in advanced disease stages at the time of confirmation [27]. Age-associated impairments in digestive and absorptive functions predispose this population to malnutrition, which undermines physiological defences against TB pathogenesis and progression [28]. Compounding these risks, insufficient social support systems – including limited familial care, suboptimal living conditions, and barriers to accessing timely and sustained treatment – further potentiate adverse health outcomes in elderly individuals with TB.

Addressing the TB disease burden in China requires a comprehensive, evidence-based strategy with continuous optimization of interventions [29, 30]. First, critical approaches to TB control include implementing active case-finding initiatives in high-risk populations and priority regions. Early detection efforts should prioritize the integration of advanced molecular and immunological diagnostic technologies to enhance case detection rates and reduce diagnostic delays. Second, optimizing therapeutic protocols to ensure sustained access to optimized treatment regimens for drug-resistant TB cases is essential, complemented by patient education and psychological support interventions to improve treatment adherence and clinical outcomes [29, 30]. Third, strengthening interdepartmental coordination across health, education, and social security sectors is fundamental for mitigating the socio-economic burden on TB patients [29, 31]. Fourth, modernizing TB surveillance systems, coupled with the development and implementation of novel vaccines, advanced diagnostics, and digital health technologies, can augment treatment adherence and longitudinal follow-up. Collectively, robust implementation of these multifaceted, dynamically optimized strategies could substantially reduce the TB disease burden in China [30, 32].

This study has several important limitations that warrant consideration. First, our estimation of TB and its subtype-specific burdens relies primarily on modelled estimates rather than direct surveillance data from China’s national infectious disease reporting infrastructure. Potential inconsistencies in clinical diagnostic accuracy, reporting proactivity, and surveillance mechanisms across regions could introduce bias in burden quantification, leading to either inflated or underestimated projections [13, 33–35]. Second, the GBD 2021 methodology for TB burden estimation incorporates limited input data dimensions, potentially compromising the validity of subtype-specific analyses [1, 12]. Third, the absence of comprehensive HIV-TB co-infection burden estimates represents a critical evidence gap in understanding intersecting epidemics. Fourth, BAPC projections for future TB burden depend heavily on historical trend extrapolation and data quality assumptions. As TB transmission dynamics are increasingly influenced by exogenous variables such as climate change and population mobility, conventional modelling approaches may inadequately capture emergent epidemiological patterns. Future burden estimations would benefit from multidimensional frameworks integrating individual-level clinical profiles, socio-economic determinants, and evolving environmental drivers to enhance predictive accuracy and policy relevance.

In conclusion, despite three decades of sustained reductions in TB burden across China, persistently elevated disease rates continue to challenge public health systems. The emerging dual threats of XDR-TB and HIV-TB co-infection have introduced additional complexity to national containment efforts. Strategic strengthening of national TB screening infrastructures will prove critical for achieving early case detection and precision therapeutic interventions. Concurrently, large-scale health literacy campaigns, community-based TB elimination programmes, and modernisation of public health facilities must form the cornerstone of a multifaceted control strategy. These coordinated measures hold potential to accelerate progress towards TB burden reduction targets while addressing systemic vulnerabilities exposed by evolving pathogen dynamics. Future initiatives should prioritize the integration of molecular surveillance, social determinant mapping, and adaptive resource allocation to sustain epidemiological gains in this transitional phase of TB control.

## Supporting information

10.1017/S0950268825100095.sm001 Zhang et al. supplementary material Zhang et al. supplementary material

## Data Availability

The data of the study are available at http://ghdx.healthdata.org/gbd-results-tool.

## Supplementary material

The supplementary material for this article can be found at http://doi.org/10.1017/S0950268825100095.
