# Supplementary material for: Trend and forecast analysis of the changing disease burden of tuberculosis in China, 1990–2021
Source: Epidemiol Infect. 2025 Jul 15;153:e85. doi: 10.1017/S0950268825100095 (PMC12322781; doi:10.1017/S0950268825100095)
Supplement: Zhang et al. supplementary material [file S0950268825100095sup001.docx]

**Supplementary**

**Trend and forecast analysis of the changing disease burden of Tuberculosis in China, 1990-2021**

Shun-Xian Zhang^1,2†^, Jin-Xin Zheng^2,3†^, Yu Wang^1^, Wen-Wen Lv^4^, Jian Yang^5^, Ji-Chun Wang^5*^ and Zhen-Hui Lu^1^*

^1^ Longhua Hospital, Shanghai University of Traditional Chinese Medicine, Shanghai 200032, China.

^2^ National Institute of Parasitic Diseases at Chinese Center for Disease Control and Prevention (Chinese Center for Tropical Diseases Research); NHC Key Laboratory of Parasite and Vector Biology; WHO Collaborating Centre for Tropical Diseases; National Center for International Research on Tropical Diseases; National Key Laboratory of Intelligent Tracking and Forecasting for Infectious Diseases, Shanghai 200025, China.

^3^ School of Global Health, Chinese Center for Tropical Diseases Research-Shanghai Jiao Tong University School of Medicine, Shanghai 200025, China.

^4^ Clinical Research Institute, Shanghai Jiao Tong University School of Medicine, Shanghai 200025, China.

^5^ Department of Science and Technology, Chinese Center for Disease Control and Prevention, Beijing 102206, China.

.

^†^ Shun-Xian Zhang and Jin-Xin Zheng contributed equally to this work.

*Corresponding authors: Ji-Chun Wang and Zhen-Hui Lu

[wangjc@chinacdc.cn;](mailto:wangjc@chinacdc.cn;) [Dr_luzh@shutcm.edu.cn](mailto:Dr_luzh@shutcm.edu.cnl)

**Items**

Figure S1: The incidence rate of HIV-negative TB and subytpes across different age groups and genders in China in 2021.

Figure S2: The prevalence rate of HIV-negative TB and subytpes across different age groups and genders in China in 2021.

Figure S3: The mortality rate of HIV-negative TB and subytpes across different age groups and genders in China in 2021.

Figure S4: The DALYs rate of HIV-negative TB and subytpes across different age groups and genders in China in 2021.

Table S1: Relationship between Tuberculosis and subtypes disease burden and SDI in China, 1990–2021.


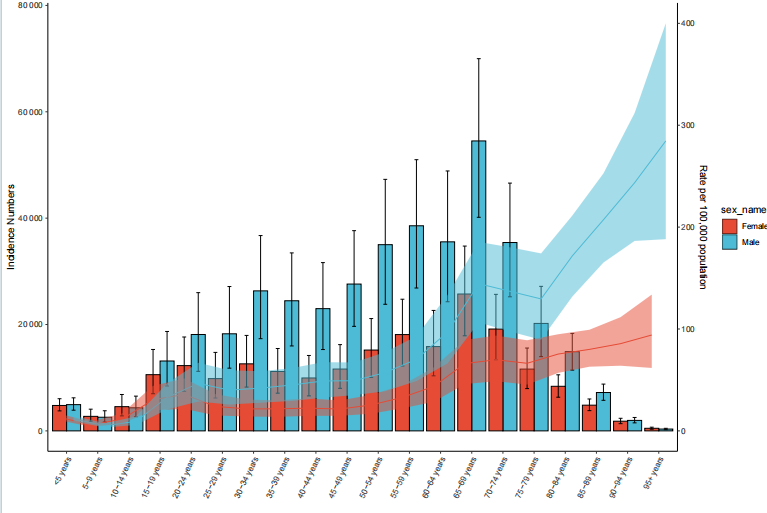

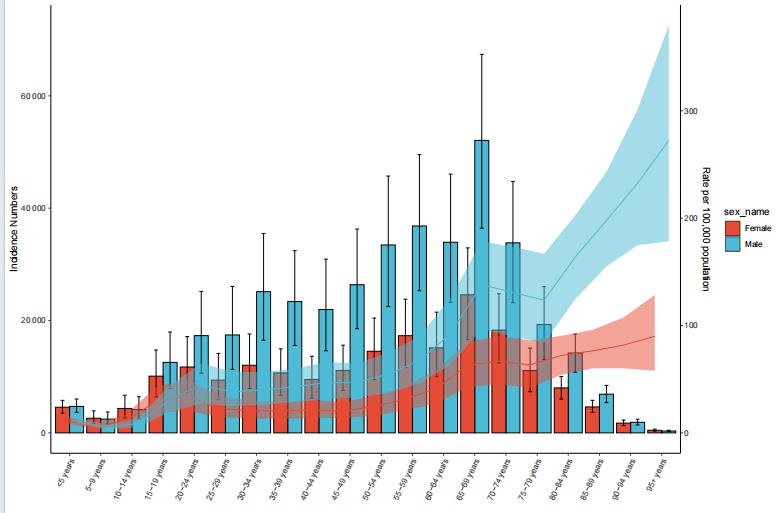

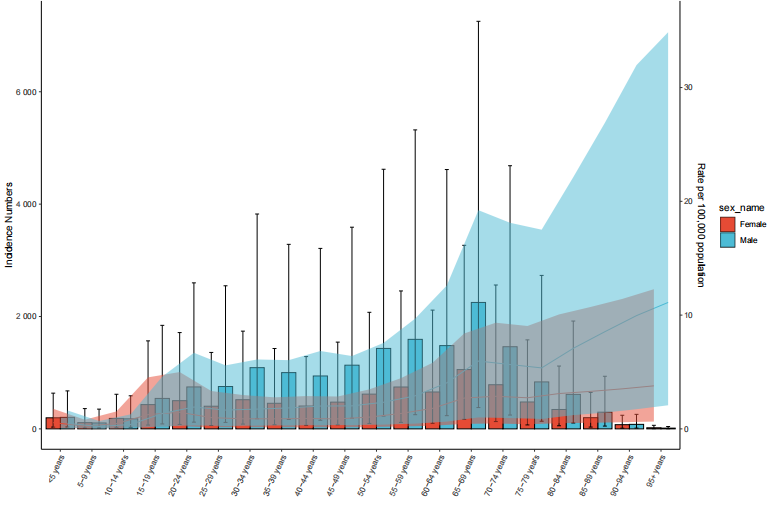

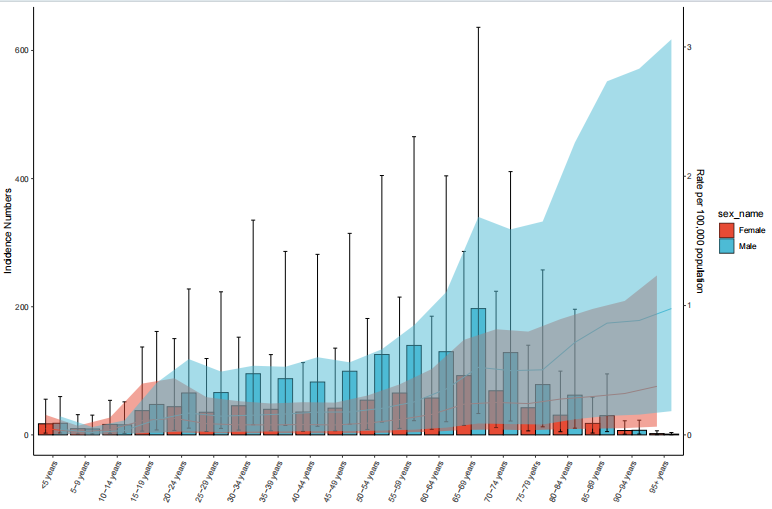


D

C

B

A


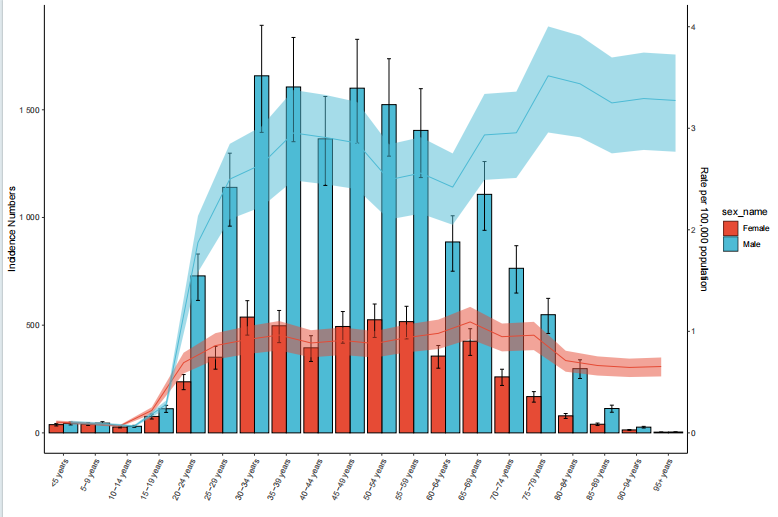

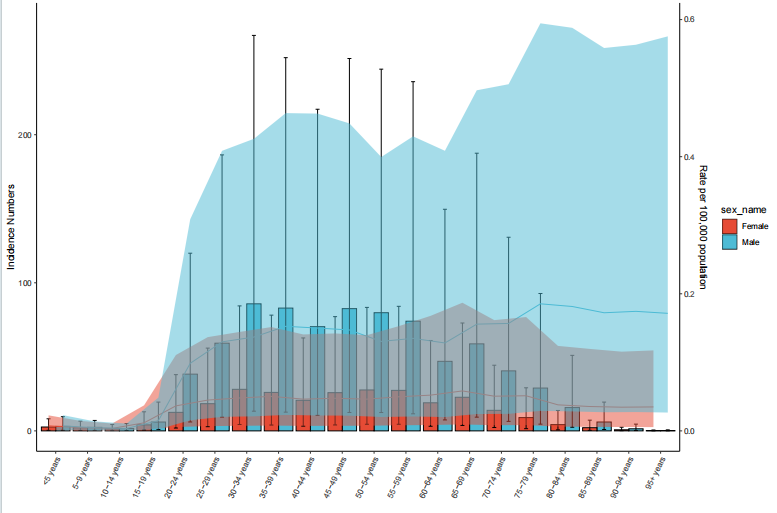


F

E


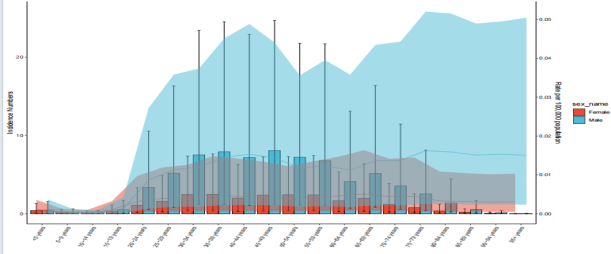


G

Figure S1: The incidence rate of HIV-negative TB and subytpes across different age groups and genders in China in 2021 (A: HIV-negative TB. B: DS-TB. C: MDR-TB. D: XDR-TB. E: HIV-DS-TB. F: HIV-MDR-TB. G: HIV-XDR-TB. Abbreviation: DS-TB=drug-susceptible tuberculosis; HIV=human immunodeficiency virus; HIV-DS-TB: HIV-infected drug-susceptible tuberculosis; HIV-MDR-TB=HIV-infected multidrug-resistant tuberculosis without extensive drug resistance; HIV-XDR-TB=HIV-infected extensively drug-resistant tuberculosis; MDR-TB=multidrug-resistant tuberculosis without extensive drug resistance; TB=Tuberculosis; XDR-TB=extensively drug-resistant tuberculosis).


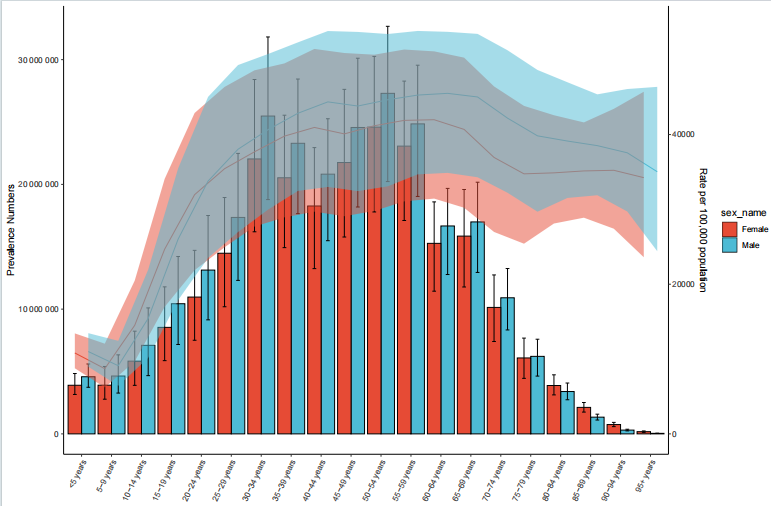

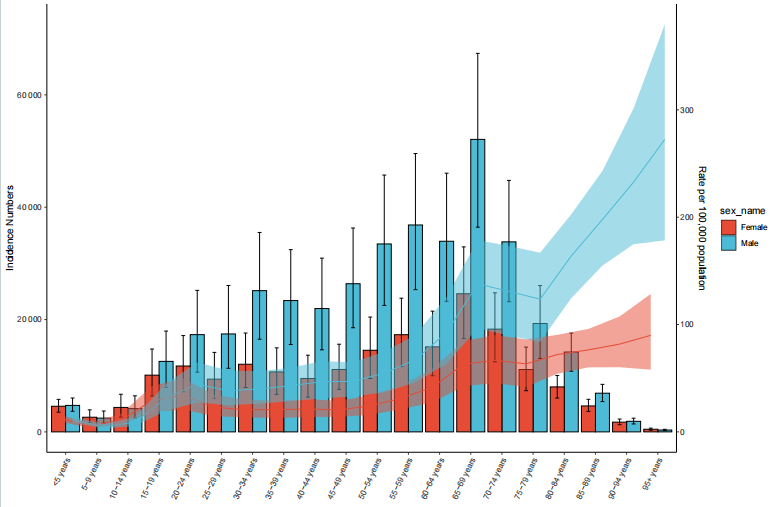


B

A


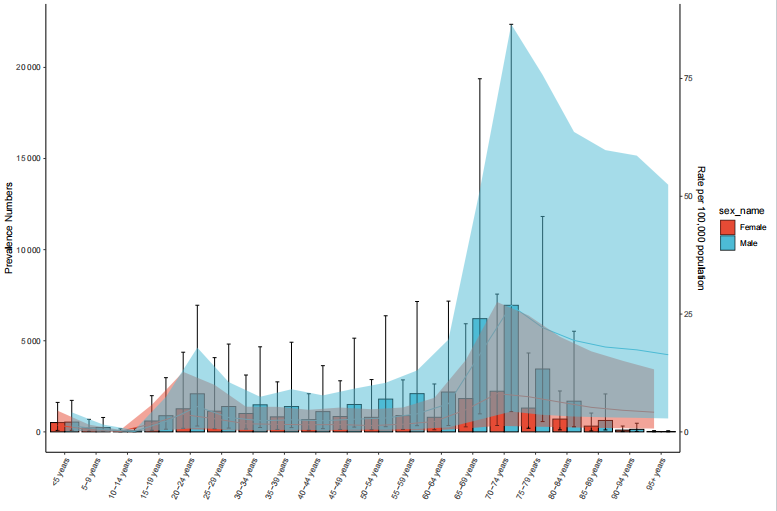

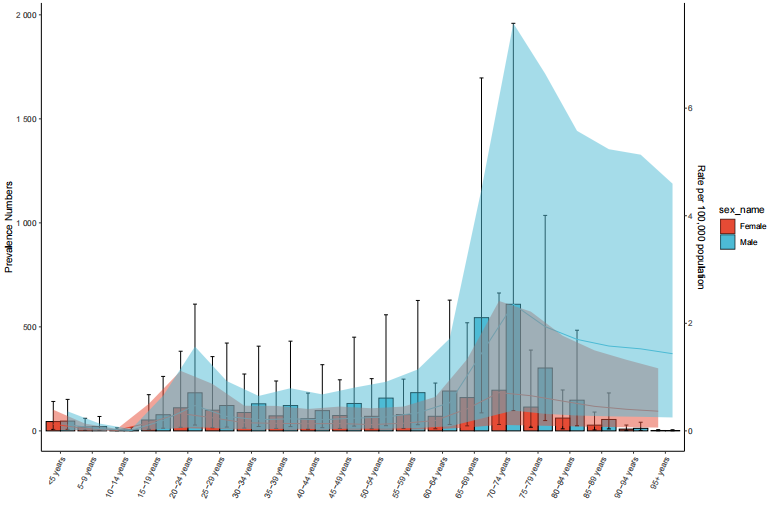


D

C


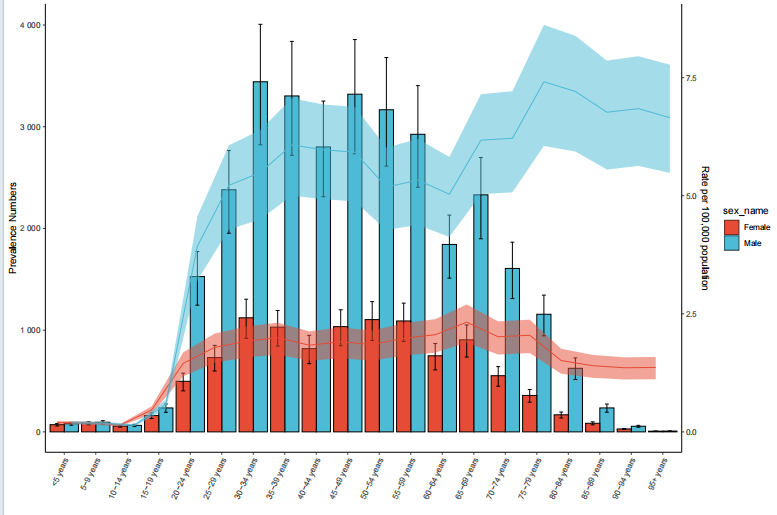

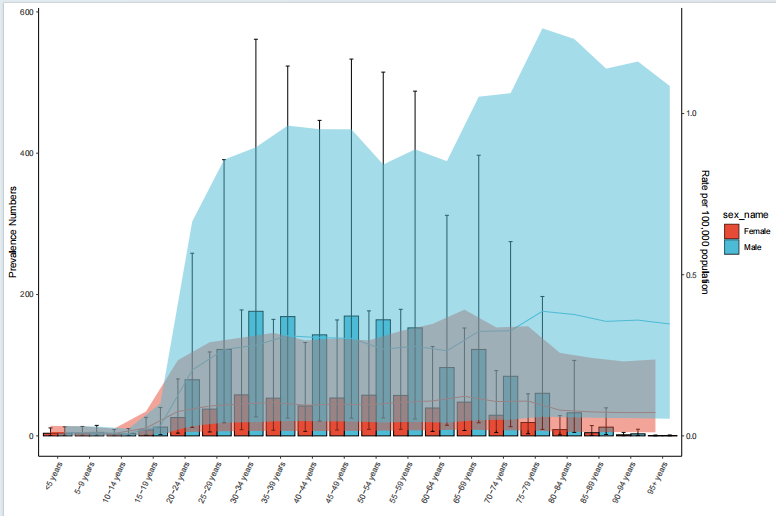


F

E


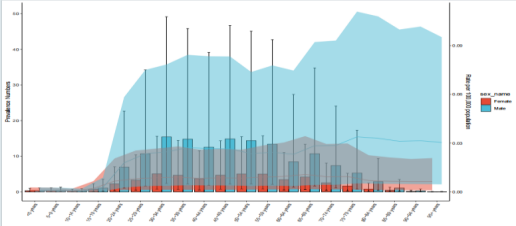


G

Figure S2: The prevalence rate of HIV-negative TB and subytpes across different age groups and genders in China in 2021 (A: HIV-negative TB. B: DS-TB. C: MDR-TB. D: XDR-TB. E: HIV-DS-TB. F: HIV-MDR-TB. G: HIV-XDR-TB. Abbreviation: DS-TB=drug-susceptible tuberculosis; HIV=human immunodeficiency virus; HIV-DS-TB: HIV-infected drug-susceptible tuberculosis; HIV-MDR-TB=HIV-infected multidrug-resistant tuberculosis without extensive drug resistance; HIV-XDR-TB=HIV-infected extensively drug-resistant tuberculosis; MDR-TB=multidrug-resistant tuberculosis without extensive drug resistance; TB=Tuberculosis; XDR-TB=extensively drug-resistant tuberculosis).


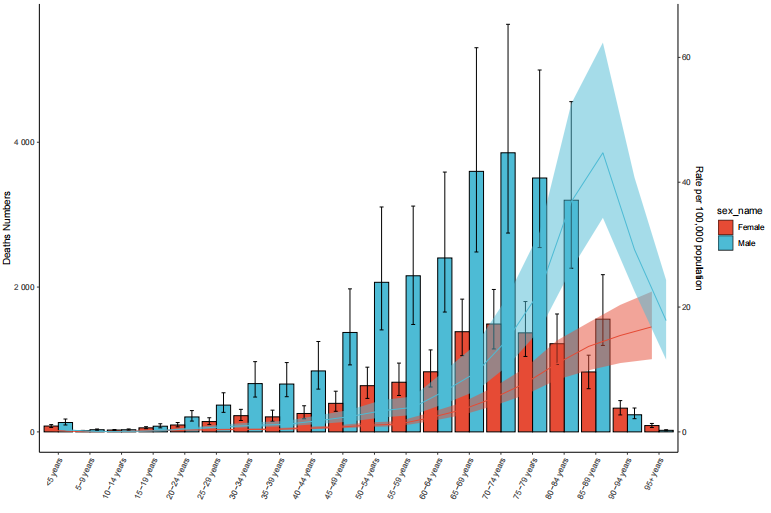

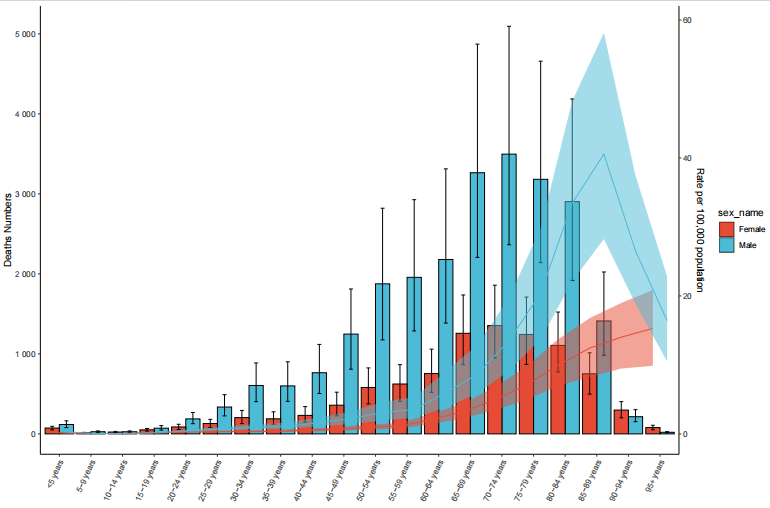


B

A


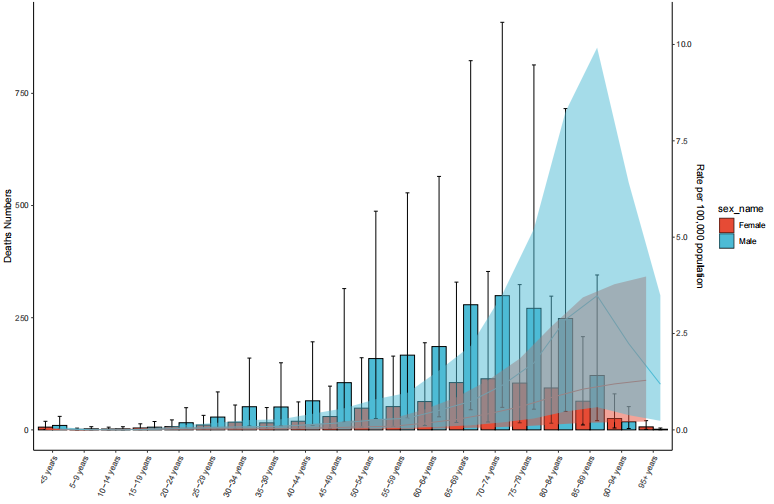

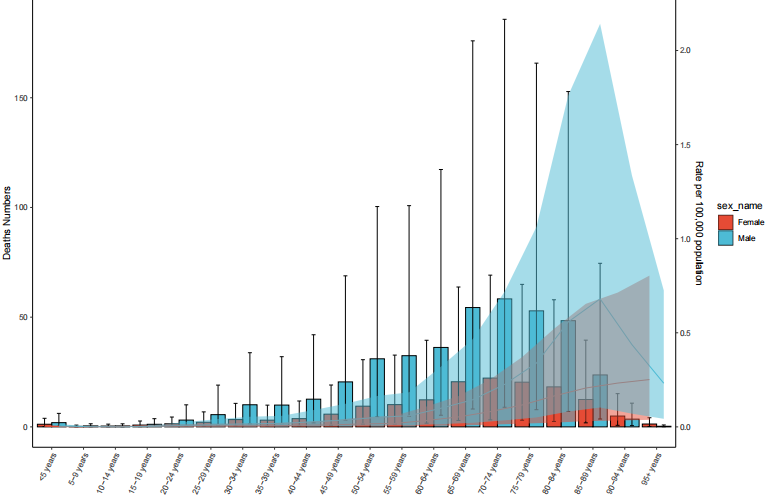


D

C


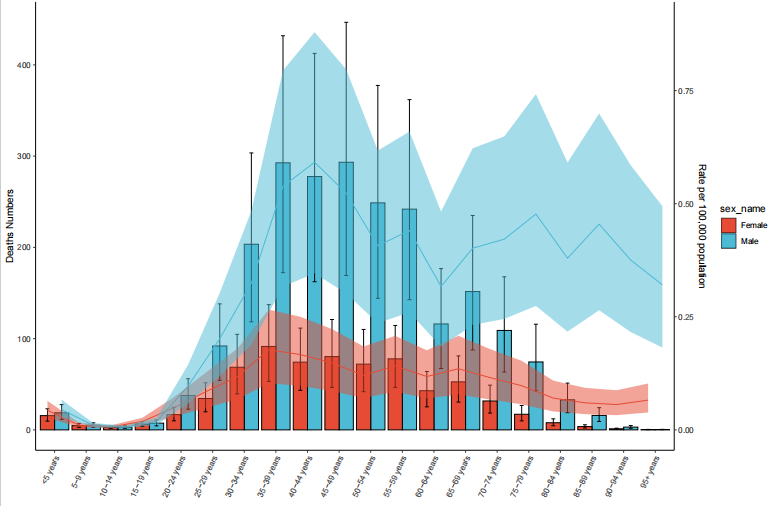

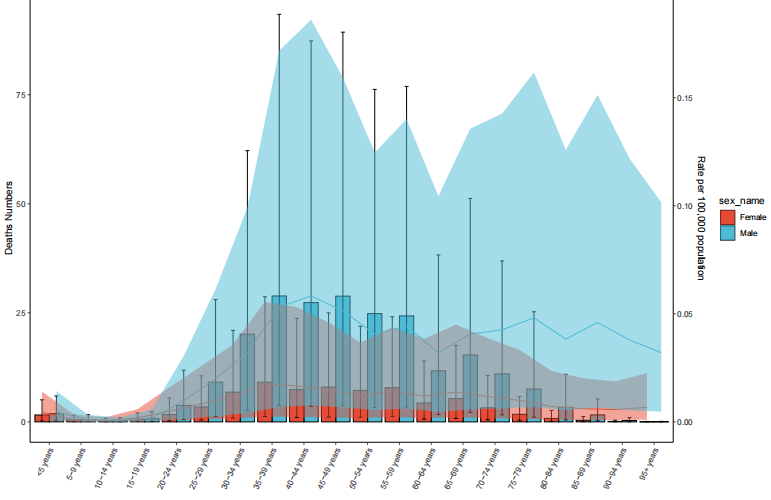


F

E


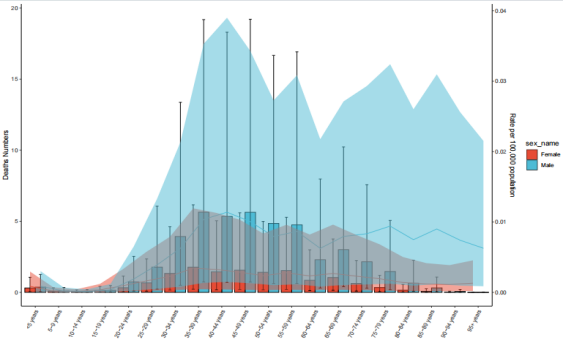


G

Figure S3: The mortality rate of HIV-negative TB and subytpes across different age groups and genders in China in 2021 (A: HIV-negative TB. B: DS-TB. C: MDR-TB. D: XDR-TB. E: HIV-DS-TB. F: HIV-MDR-TB. G: HIV-XDR-TB. Abbreviation: DS-TB=drug-susceptible tuberculosis; HIV=human immunodeficiency virus; HIV-DS-TB: HIV-infected drug-susceptible tuberculosis; HIV-MDR-TB=HIV-infected multidrug-resistant tuberculosis without extensive drug resistance; HIV-XDR-TB=HIV-infected extensively drug-resistant tuberculosis; MDR-TB=multidrug-resistant tuberculosis without extensive drug resistance; TB=Tuberculosis; XDR-TB=extensively drug-resistant tuberculosis).


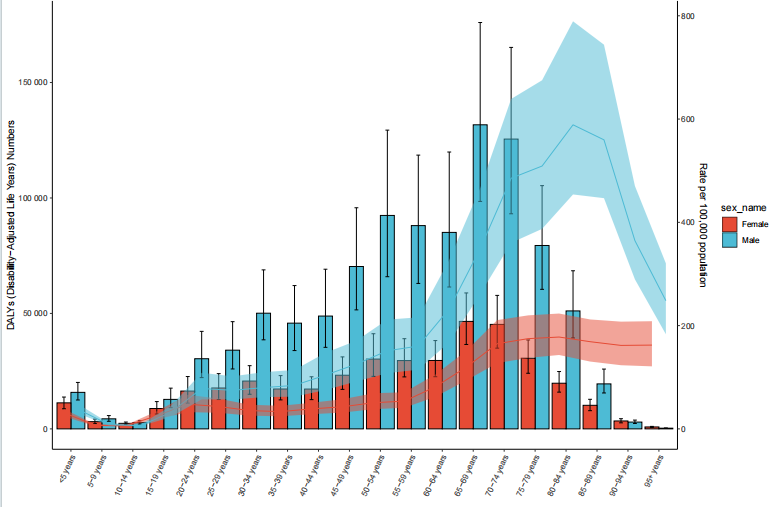

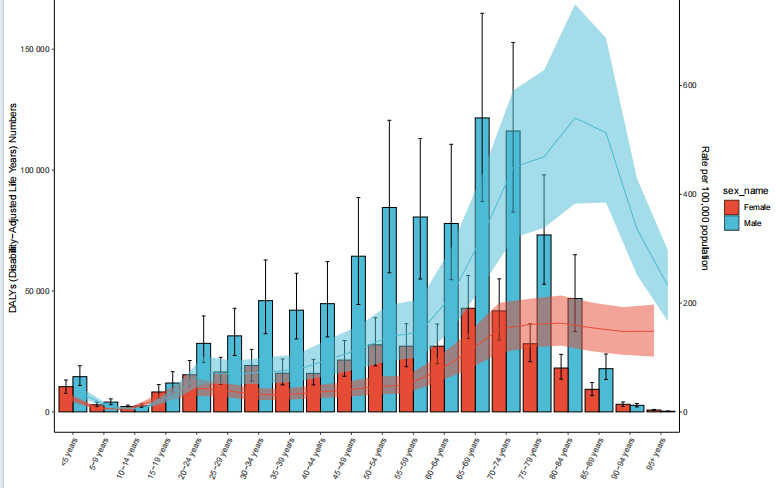


B

A


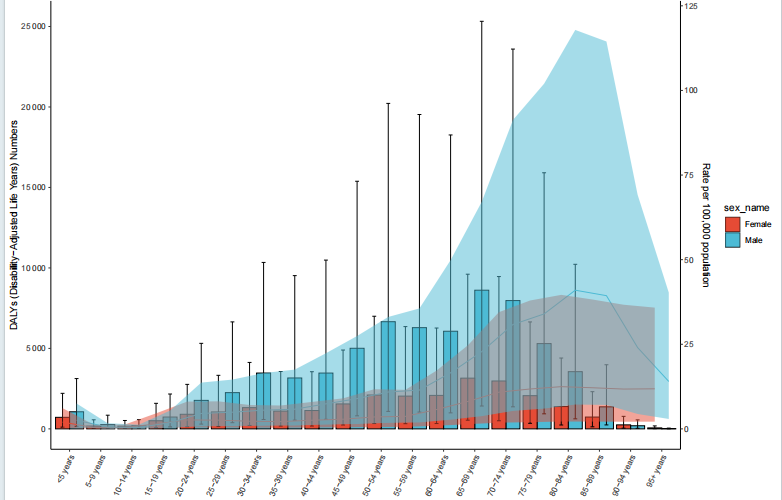

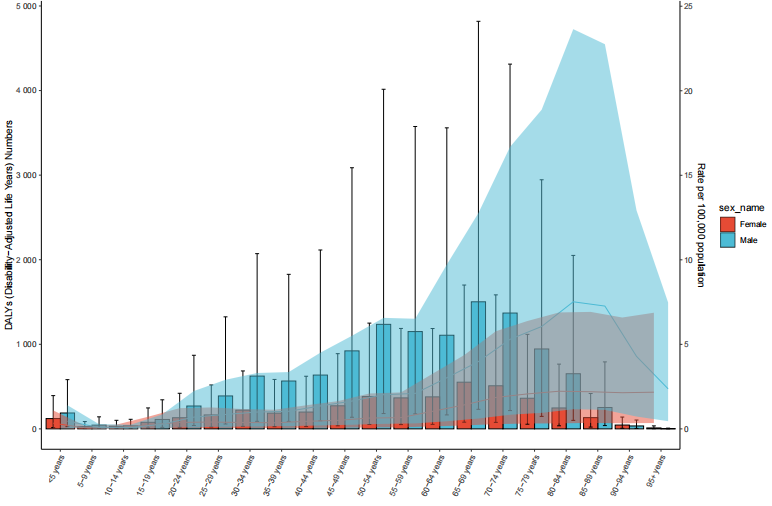


D

C


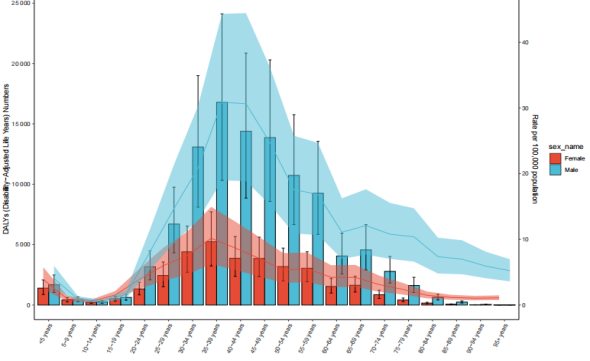

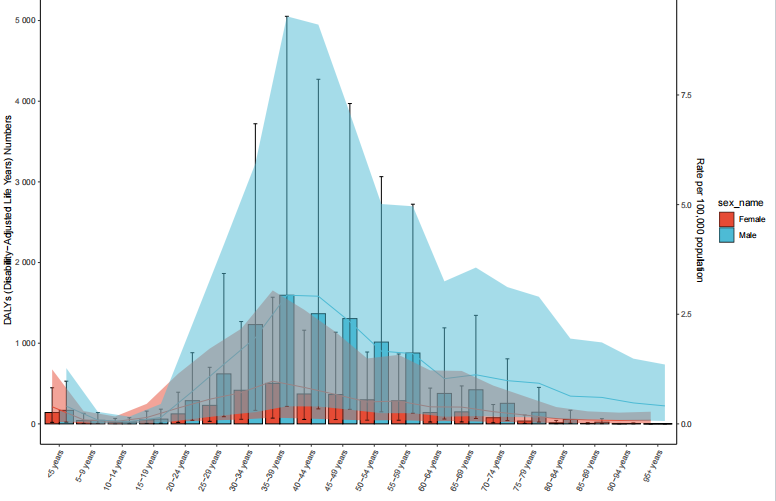


F

E


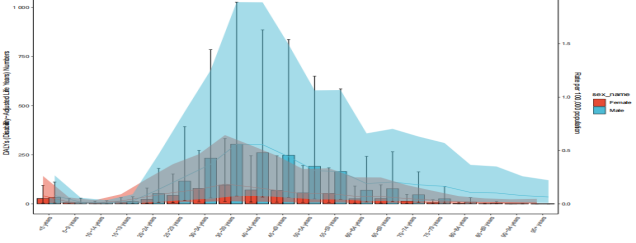


G

Figure S4: The DALYs rate of HIV-negative TB and subytpes across different age groups and genders in China in 2021 (A: HIV-negative TB. B: DS-TB. C: MDR-TB. D: XDR-TB. E: HIV-DS-TB. F: HIV-MDR-TB. G: HIV-XDR-TB. Abbreviation: DALY=Disability-adjusted life year. DS-TB=drug-susceptible tuberculosis; HIV=human immunodeficiency virus; HIV-DS-TB: HIV-infected drug-susceptible tuberculosis; HIV-MDR-TB=HIV-infected multidrug-resistant tuberculosis without extensive drug resistance; HIV-XDR-TB=HIV-infected extensively drug-resistant tuberculosis; MDR-TB=multidrug-resistant tuberculosis without extensive drug resistance; TB=Tuberculosis; XDR-TB=extensively drug-resistant tuberculosis).

Table S1: Relationship between Tuberculosis and subtypes disease burden and SDI in China, 1990–2021

| Disease | Index | *r* | *P* |
| --- | --- | --- | --- |
| Tuberculosis | ASIR | -0.998 | <0.001 |
| Tuberculosis | ASPR | -0.696 | <0.001 |
| Tuberculosis | ASMR | -0.999 | <0.001 |
| Tuberculosis | Age-standardized DALY rate | -0.999 | <0.001 |
| HIV/AIDS | ASIR | 0.335 | 0.061 |
| HIV/AIDS | ASPR | 0.900 | <0.001 |
| HIV/AIDS | ASMR | 0.958 | <0.001 |
| HIV/AIDS | Age-standardized DALY rate | 0.973 | <0.001 |
| DS-TB | ASIR | -0.999 | <0.001 |
| DS-TB | ASPR | -0.999 | <0.001 |
| DS-TB | ASMR | -0.999 | <0.001 |
| DS-TB | Age-standardized DALY rate | -0.999 | <0.001 |
| MDR-TB | ASIR | -0.868 | <0.001 |
| MDR-TB | ASPR | -0.835 | <0.001 |
| MDR-TB | ASMR | -0.937 | <0.001 |
| MDR-TB | Age-standardized DALY rate | -0.937 | <0.001 |
| XDR-TB | ASIR | 0.029 | 0.875 |
| XDR-TB | ASPR | 0.215 | 0.244 |
| XDR-TB | ASMR | -0.541 | 0.003 |
| XDR-TB | Age-standardized DALY rate | -0.284 | 0.122 |
| HIV-DS-TB | ASIR | 0.642 | <0.001 |
| HIV-DS-TB | ASPR | 0.848 | <0.001 |
| HIV-DS-TB | ASMR | 0.374 | 0.036 |
| HIV-DS-TB | Age-standardized DALY rate | 0.424 | 0.016 |
| HIV-MDR-TB | ASIR | -0.481 | 0.006 |
| HIV-MDR-TB | ASPR | -0.260 | 0.151 |
| HIV-MDR-TB | ASMR | -0.263 | 0.146 |
| HIV-MDR-TB | Age-standardized DALY rate | -0.244 | 0.178 |
| HIV-XDR-TB | ASIR | 0.508 | 0.004 |
| HIV-XDR-TB | ASPR | 0.793 | <0.001 |
| HIV-XDR-TB | ASMR | 0.521 | 0.002 |
| HIV-XDR-TB | Age-standardized DALY rate | 0.464 | 0.009 |

Abbreviation: ASIR=age-standardized incidence rate; ASPR=age-standardized prevalence rate; ASMR=age-standardized mortality rate; DALY=Disability-adjusted life year. DS-TB=drug-susceptible tuberculosis; HIV/AIDS=human immunodeficiency virus/Acquired Immune Deficiency Syndrome; HIV-DS-TB: HIV-infected drug-susceptible tuberculosis; HIV-MDR-TB=HIV-infected multidrug-resistant tuberculosis without extensive drug resistance; HIV-XDR-TB=HIV-infected extensively drug-resistant tuberculosis; MDR-TB=multidrug-resistant tuberculosis without extensive drug resistance; TB=Tuberculosis; XDR-TB=extensively drug-resistant tuberculosis.
